# Rutin prevents tau pathology and neuroinflammation in a mouse model of Alzheimer’s disease

**DOI:** 10.1186/s12974-021-02182-3

**Published:** 2021-06-11

**Authors:** Xiao-ying Sun, Ling-jie Li, Quan-Xiu Dong, Jie Zhu, Ya-ru Huang, Sheng-jie Hou, Xiao-lin Yu, Rui-tian Liu

**Affiliations:** 1 State Key Laboratory of Biochemical Engineering, Institute of Process Engineering, Chinese Academy of Sciences, Beijing, 100190 China; 2 Innovation Academy for Green Manufacture, Chinese Academy of Sciences, Beijing, 100190 China; 3 School of Chemistry and Chemical Engineering, University of Chinese Academy of Science, Beijing, 100049 China

**Keywords:** Alzheimer’s disease, Tau pathology, Neurofibrillary tangles, Neuroinflammation, Synapse loss, Rutin

## Abstract

**Background:**

Tau pathology is a hallmark of Alzheimer’s disease (AD) and other tauopathies. During disease progression, abnormally phosphorylated forms of tau aggregate and accumulate into neurofibrillary tangles, leading to synapse loss, neuroinflammation, and neurodegeneration. Thus, targeting of tau pathology is expected to be a promising strategy for AD treatment.

**Methods:**

The effect of rutin on tau aggregation was detected by thioflavin T fluorescence and transmission electron microscope imaging. The effect of rutin on tau oligomer-induced cytotoxicity was assessed by MTT assay. The effect of rutin on tau oligomer-mediated the production of IL-1β and TNF-α in vitro was measured by ELISA. The uptake of extracellular tau by microglia was determined by immunocytochemistry. Six-month-old male Tau-P301S mice were treated with rutin or vehicle by oral administration daily for 30 days. The cognitive performance was determined using the Morris water maze test, Y-maze test, and novel object recognition test. The levels of pathological tau, gliosis, NF-kB activation, proinflammatory cytokines such as IL-1β and TNF-α, and synaptic proteins including synaptophysin and PSD95 in the brains of the mice were evaluated by immunolabeling, immunoblotting, or ELISA.

**Results:**

We showed that rutin, a natural flavonoid glycoside, inhibited tau aggregation and tau oligomer-induced cytotoxicity, lowered the production of proinflammatory cytokines, protected neuronal morphology from toxic tau oligomers, and promoted microglial uptake of extracellular tau oligomers in vitro. When applied to Tau-P301S mouse model of tauopathy, rutin reduced pathological tau levels, regulated tau hyperphosphorylation by increasing PP2A level, suppressed gliosis and neuroinflammation by downregulating NF-kB pathway, prevented microglial synapse engulfment, and rescued synapse loss in mouse brains, resulting in a significant improvement of cognition.

**Conclusion:**

In combination with the previously reported therapeutic effects of rutin on Aβ pathology, rutin is a promising drug candidate for AD treatment based its combinatorial targeting of tau and Aβ.

**Supplementary Information:**

The online version contains supplementary material available at 10.1186/s12974-021-02182-3.

## Background

Alzheimer’s disease (AD) is an age-related neurodegenerative disorder characterized by progressive memory loss [1], the deposition of extracellular Aβ plaques, and intracellular neurofibrillary tangles (NFTs) composed of tau aggregates [2, 3]. Tau is a microtubule (MT)-associated protein predominantly expressed in neuronal axons with a primary function of promoting assembly and stability of MTs [4]. Clinical observations and insights implicate that NFTs are closely link to synapse loss, gliosis, neuroinflammation, and clinical symptoms including cognitive impairment and neurodegeneration [5, 6].

During procession of AD and other tauopathies, hyperphosphorylation of tau is one of the earliest and continuous events, which disrupt the association of tau with MTs, and promote tau aggregation in neurons and relocation to synapses [7, 8]. Pathological tau may destroy synaptic composition and structure, leading to synaptic dysfunction and subsequent synapse loss [9, 10]. Tau oligomers have emerged as the most toxic species in tauopathies, which cause the spreading of tau pathology, induce further tau aggregation and neuroinflammation [11]. Each stage in tau pathology, from tau expression to post-translational modifications and aggregation, or tau-induced synapse loss and neuroinflammation, presents opportunities for intervention [12]. To date, various potential tau-targeting therapies, including small-molecule therapies and immunotherapies, have reached the clinical trial stage for AD and other tauopathies. However, none of them has yet been successfully translated into benefits in humans, partly because of poor efficacy or side effects [8]. Given tau pathology is a complex multifactorial process, strategies targeting multiple phases for therapeutic intervention might be promising.

Many natural products have shown progresses toward AD treatment, due to their neuroprotective effects and good safety profile. Rutin is a natural flavonoid glycoside with diverse biological effects, such as anti-microbial, anti-carcinogenic, anti-thrombotic, cardioprotective, and neuroprotective activities [13]. These biological functions are mainly associated with its anti-inflammatory and antioxidant activities [14]. As a neuroprotective agent, rutin exerts beneficial effects on various neurodegenerative diseases, including AD, Parkinson’s disease (PD), and Huntington’s disease (HD) [15]. Pharmacological studies indicate that rutin and its derivates protect dopaminergic neurons, mitigate apoptosis, astrogliosis, and oxidative stress in 6-hydroxydopamine-induced rat model of PD [16, 17]. Rutin is recently reported to reverse HD through insulin/IGF1 (IIS) signaling pathway and autophagy activity [18]. For AD, rutin has also been studied in several models of Aβ-related pathology [14, 19–21]. In our previous studies, we found that rutin improved spatial memory and reduced Aβ oligomer level and neuroinflammation in APP/PS1 mouse model of AD [14].

In this study, we investigated the effects of rutin on tau aggregation and cytotoxity, tau oligomer-induced the production of proinflammatory cytokines, as well as microglial engulfment of extracellular tau in vitro, and assessed its therapeutic effect on cognitive performance and neuropathology in Tau-P301S mouse model of tauopathy.

## Methods

### Preparation of tau oligomers and rutin stock solution for in vitro study

The recombinant human wild-type 2N4R tau protein expression vector was a kind gift from Dr. Virginia M.-Y. Lee. Tau proteins were expressed in *Escherichia coli* and purified by heat denaturation followed by cation exchange chromatography [22]. Tau oligomers were prepared by dissolving tau proteins in aggregation buffer (20 mM Tris-HCl, 150 mM NaCl, 2.5 μM heparin, pH 8.0) to 10 μM and incubating at 37 °C with agitation for 24 h, and then separated by size exclusion chromatography (Fig. S1a). There are two main kinds of soluble oligomer species present in our tau oligomer preparations. The smaller oligomers are about 600–720 kDa, and the larger oligomers are about 1000–1200 kDa. Their oligomeric states were confirmed using western blotting (Fig. S1b). For phagocytosis studies, a final concentration of 1 μM tau oligomers was used.

For rutin stock solution, rutin (China National Institutes for Food and Drug Control, R5143-1G, ≥ 98%) was dissolved in DMSO to 32 mM and diluted with PBS (pH 7.4) to the indicated concentrations.

### Thioflavin T fluorescence assay

To monitor tau aggregation kinetics, tau proteins (monomers) were dissolved in aggregation buffer (20 mM Tris-HCl, 150 mM NaCl, 2.5 μM Heparin, pH 8.0) to 10 μM and incubating at 37 °C with agitation. Thioflavin T (ThT, Sigma) was dissolved in 50 mM phosphate buffer (pH 6.5) to a final concentration of 5 μM. The ThT fluorescence intensity of tau sample was measured by adding 10 μL aliquot of the sample to 190 μL ThT solution in a 96-well black plate with a Tecan Safire2 microplate reader (Tecan, Switzerland) set to 450 nm/482 nm (excitation/emission). The experiments were performed three times with at least biological triplicates in each experiment.

### Transmission electron microscopy (TEM) imaging

TEM was used for the morphological examination of tau. Briefly, tau samples (10 μL) were spotted onto a 200-mesh formvar-coated copper grid (Electron Microscopy Sciences, Hatfield, PA, USA) for 5 min. The grid was then stained with 10 μL of 2% uranyl acetate for 30 s, and blotted with deionized water and air-dried at room temperature. The samples were detected using a Hitachi H7650 TEM system (Hitachi, Japan) at 80 kV with a × 60,000 magnification.

### MTT assay

SH-SY5Y neuroblastoma cells were maintained in Dulbecco’s modified Eagle’s medium (DMEM/High Glucose, Hyclone) containing 10% fetal bovine serum (FBS) and 1% penicillin/streptomycin at 37 °C under a 5% CO_2_ atmosphere. The cells were seeded in 96-well plates with approximately 7500 cells per 100 μL of medium per well. Plates were incubated at 37 °C for 24 h to allow the cells to attach. One μM tau oligomers with or without different concentrations of rutin was then added to the cell cultures and the plates were incubated for an additional 72 h at 37 °C. Cell viability was determined by adding 25 μL of 5 mg/mL MTT to each well. After 3 h of incubation at 37 °C, the medium was gently removed, and a 150-μL aliquot of DMSO was added to each well. Plates were then shaken at room temperature for 10 min and the absorbance at 570/630 nm was measured by using a SpectraMax M5 microplate reader (Molecular Devices, Sunnyvale, CA). Data were obtained from three independent experiments with at least biological triplicates in each experiment.

### Primary mouse neurons and microglia

Primary neurons were obtained from hippocampi of mouse embryos on embryonic day 16(E16) to E17. Briefly, Hippocampi were dissected, trypsinized, and triturated, and the resulting dissociated cells were plated on poly-D-lysine-coated coverslips at a density of 300,000/well in 12-well dish and cultured in neurobasal medium with B27 and l-GlutaMAX. Half of the medium was exchanged with fresh medium three times a week. Primary microglia were obtained from cortices and hippocampi from postnatal (P1–P2) pups of mice. Cells were cultured in DMEM with 10% FBS in 75-cm^2^ flasks for 12 days. Loosely attached microglia were harvested at DIV12 by shaking for 60 min at 180 rpm.

### Immunocytochemistry staining and image analysis

Primary neurons and microglial cells were exposed to tau oligomers (1 μM) in the absence or presence of rutin (8 μM) for 24 h. After that, cells were rinsed three times in PBS, fixed in 4% paraformaldehyde for 20 min at room temperature, permeabilized with 0.3% Triton X-100 for 25 min, and blocked with 10% donkey serum albumin (DSA) for 30 min. Then cells were processed for immunofluorescence using anti-MAP2 (Abcam, ab32454, 1:100), HT-7 (Thermo fisher, MN1000, 1:100) and anti-Iba-1 (GeneTex, GTX101495, 1:100) antibodies for 1 h at room temperature, followed by corresponding secondary antibodies conjugated to Alexa Fluor 488 (Abcam, ab150081, 1:300) or Alexa Fluor 594 (Abcam, ab150120, 1:300), respectively. Subsequently, cells were incubated with Hoechst (1:10000) for 15 min in the dark and mounted on coverslips with anti-fade mounting medium (Solarbio, China). Fluorescence signals were captured on a laser scanning confocal microscope (Leica TCS SP8, Germany). Sholl analysis was performed using an ImageJ plugin [23], briefly, thresholded bitmap images were measured from soma to furthest dendrite in the MAP2 channel. Then concentric circles of gradually increasing (20 μm) radii were calculated, and the crossing points with the MAP2 channel automatically counted. Forty neurons per culture from three independent cultures were used for the analysis.

For microglial engulfment analysis, immunostained microglial cells were imaged on a Leica TCS SP8 confocal microscope taking 10–12 z-stacks (0.5 μm step size). Maximum intensity projections were created, and the Iba-1 fluorescence signal was thresholded and used as a mask to detect HT7-positive tau puncta in microglial cells. All puncta within the microglial cells were examined. At least 20 microglial cells per culture from three independent cultures were used for the analysis.

### Mice and rutin administration

Six-month-old male Tau-P301S mice were originally obtained from Jackson Laboratory (stock No.008169), which express the P301S mutant form of human tau, exhibiting many neuropathological features of human tauopathies including cognitive deficits, gliosis, neuroinflammation, and synapse loss. Non-transgenic littermates were used as controls. All mice for experiments were group-housed, provided food and water ad libitum, and kept in a colony room at 22 ± 2 °C and 45% ± 10% humidity on a reverse 12-h light/dark cycle. All experiments were performed in accordance with the China Public Health Service Guide for the Care and Use of Laboratory Animals. Experiments involving mice and protocols were approved by the Institution Animal Care and Use Committee of Tsinghua University. All mice were randomly treated in cohorts. Rutin was suspended in 0.5% carboxymethylcellulose (CMC) to 10 mg/mL. All mice were treated with rutin (100 mg/kg) or vehicle (0.5% CMC) daily by orally administration (100 μL rutin suspension or vehicle per 10 g body weight) for 30 days. Tau-P301S mice were categorized into two groups by treatment: rutin (Tg-Rutin) and vehicle (Tg-Veh). Their WT littermates were categorized into two groups: rutin (WT-Rutin) and vehicle (WT-Veh). After the last administration, all mice were tested for preferences and motor function. Eight mice without preferences and motor dysfunction in every group completed each behavioral task.

### Morris water maze (MWM) test

The water maze consisted of a pool (110 cm in diameter) containing opaque water (22 ± 1 °C) and a platform (10 cm in diameter) submerged 1.0 cm under the water. During the training trial, mice were allowed to swim for 60 s to locate the hidden platform, and they were allowed to stay on it for 10 s upon finding the platform. Mice unable to locate the platform were guided to it. The mice were trained twice per day over five consecutive days, with an inter-trial interval of 3–4 h. Twenty-four hours after the last training trial, the mice were tested for memory retention in a probe trial in the absence of the platform. The performance of each mouse was monitored using a video camera (Sony, Tokyo, Japan) mounted over the maze and automatically recorded via a video tracking system.

### Y-maze test

The Y-maze apparatus consisted of three arms (8 × 30 × 15 cm) separated by an angle of 120°.The Y-maze test consisted of two trials separated by an interval of 1 h. The first trial was in a 10-min duration. Mice were allowed to explore only two arms (start arm and familiar arm) of the maze, with the third arm (novel arm) being blocked. In the second trial, mice were put back in the maze in the starting arm, with free access to all three arms for 5 min. The total time spent in the novel arm was recorded using a ceiling-mounted camera and analyzed.

### Novel objection recognition (NOR) test

The NOR test is based on the spontaneous tendency of mice to exhibit more interactions with a novel rather than a familiar object. Briefly, in the habituation phase, mice were allowed freely exploring the behavioral arena (50 cm × 50 cm × 25 cm white plastic box, empty) for 5 min 1 day before testing. For training session, mice were placed in the box having two identical objects in the upper two corners and allowed to explore for 5 min. After a 24-h retention period, in the testing session, the right object was replaced with a novel object in the original location, and the mice were reintroduced to the box and allowed to explore for 5 min. The time spent exploring and sniffing each object was recorded. The results are expressed as the discrimination index, which refers to: $\frac{\mathrm{Time}\ \mathrm{novel}-\mathrm{Time}\ \mathrm{familiar}}{\mathrm{Time}\ \mathrm{novel}+\mathrm{Time}\ \mathrm{familiar}}$. The box was cleaned with 70% alcohol between trials to eliminate olfactory cues.

### Immunohistochemistry and image analysis

Mice were deeply anesthetized with avertin and transcardially perfused with ice-cold PBS containing heparin (10 U/mL) before sacrificed. Their brains were immediately removed and divided along the sagittal plane. The left brain hemisphere was fixed in 4% paraformaldehyde in PBS at 4 °C overnight and processed for paraffin-embedded sections. For immunostaining, 5 μm of coronal serial sections were deparaffinized and subjected to antigen retrieval using citrate buffer (0.01 M, pH 6.0) at 95 °C for 20 min. The sections were then incubated with 3% H_2_O_2_ and washed 3 times with 1× PBS. Sections were then permeabilized and blocked with 10% normal goat serum in 0.3% Triton X-100 PBST for 1 h at room temperature. Then sections were immunostained with anti-Iba-1 (GeneTex, GTX101495, 1:100), anti-GFAP (Cell Signaling Technology, 3670S, 1:100), or anti-AT8 (ThermoFisher, MN1020, 1:100) antibodies, followed by appropriate HRP-labeled secondary antibodies and visualized with diaminobenzidine (DAB). For synaptic protein staining, sections were immunostained with anti-synaptophysin (Abcam, ab32127, 1:100) and anti-PSD95 (Abcam, ab12093, 1:100) antibodies followed by corresponding secondary antibodies conjugated to Alexa Fluor 488 (Santa Cruz, I1112, 1:1000) or Alexa Fluor 594 (Abcam, ab150084, 1:1000), respectively. All images were acquired with an Olympus IX73 inverted microscope with DP80 camera or Leica TCS SP8 confocal microscope. Three to seven coronal sections spanning the cortex and hippocampus at different depths were analyzed for each animal. Six images were acquired on matching areas per section. For AT8-, GFAP-, and Iba-1 immunostaining, the positive DAB-staining area was quantified. For immunofluorescence analysis of synaptic proteins, the mean fluorescence signal intensity was measured. Values were normalized to the mean value of vehicle-treated Tau-P301S mice (Tg-Veh) and expressed as percentage means ± SEM. All images were analyzed by ImageJ Software (National Institutes of Health, USA). The experimenter was blinded to the treatment group.

To analyze the colocalization of pre- and postsynaptic markers, the pre- and postsynaptic puncta were considered as colocalized if at least one pixel of synaptophysin puncta colocalized with a PSD-95 cluster.

For microglial engulfment assays, sections were incubated with anti-Iba-1 and anti-PSD95 antibodies overnight at 4 °C, followed by corresponding secondary antibodies conjugated to Alexa Fluor 488 or 647. The brain sections were imaged on a Leica TCS SP8 confocal microscope taking up to 55–60 z-stacks at 0.2 μm steps. At least 20 microglia within the hippocampal CA1 region were randomly chosen for each mouse. The individual images of microglia were processed and analyzed in Imaris software. The total volume of engulfed inputs in microglia was quantified. Image acquisition, quantification, and analyses were performed blind.

### Brain lysate preparation and western blot

The right brain hemisphere was homogenized in a modified RIPA buffer supplemented with complete protease inhibitor mixture tablets (Sigma, P2714-1BTL), followed by centrifugation at 12,000×*g* for 30 min at 4 °C. The supernatants were obtained and the protein concentrations were determined using the BCA protein assay (Pierce). Protein samples were separated by a 4–12% SDS-PAGE gel (Invitrogen) and transferred onto nitrocellulose membranes. After blocking with 5% non-fat milk for 2 h at room temperature, the membrane was probed with AT8 (Thermo, MN1020, 1:1000), anti-Iba-1 (Gene Tex, GTX101495, 1:1000), anti-GFAP (CST, 3670S, 1:1000), anti-PP2A (abcam, ab32104), anti-p-P65 (CST, 13346S, 1:1000), anti-P65 (Santa Cruz, sc-7151, 1:1000), anti-IKKβ (CST, 2678S, 1:1000), and anti-β-actin (MBL, M177-3, 1:1000), respectively, followed by appropriate HRP-conjugated secondary antibodies. Bands in immunoblots were developed with Super-Signal West Pico Plus Chemiluminescent Substrate kit (Pierce, UB278521), and quantified by densitometry using ImageJ software (NIH).

### Dot-blot

One microgram (0.5 μg/μL) of the brain lysates was applied to nitrocellulose membranes. The membrane was blocked with 5% non-fat milk in TBST and incubated with OC (Millipore, AB2286, 1:1000) or anti-β-actin (MBL, M177-3, 1:1000) antibodies for 1 h at room temperature, followed by appropriate HPR-conjugated secondary antibodies. Immuno-reactive blots were developed with Super-Signal West Pico Plus Chemiluminescent Substrate kit (Pierce, UB278521), and quantified by densitometry using ImageJ software (NIH).

### Measurement of IL-1β and TNF-α

For in vitro study, primary microglial cells were exposed to tau oligomers (1 μM) in the absence or presence of rutin (8 μM) for 24 h. The levels of IL-1β and TNF-α in the supernatant of cell cultures were determined using ELISA kits (Neobioscience technology, Beijing, China), according to the manufacturer’s protocols. The absorbance of the reactions was measured at 450 nm using SpectraMax M5 microplate reader (Molecular Devices, Sunnyvale, CA). For in vivo study, the levels of IL-1β and TNF-α in the brain lysates of mice were determined using the same protocol.

### Statistical analysis

Data were analyzed with GraphPad Prism v.8. Statistical significance was assessed using student’s *t* test, one-way or two-way ANOVA followed by Tukey’s test or Bonferroni’s test. Results were expressed as group mean ± SEM, and P < 0.05 was considered statistically significant. All samples or animals were included for statistical analysis unless otherwise noted in pre-established criteria.

## Results

### Rutin inhibits tau aggregation and reduces tau-mediated cytotoxicity

**Fig. 1:**
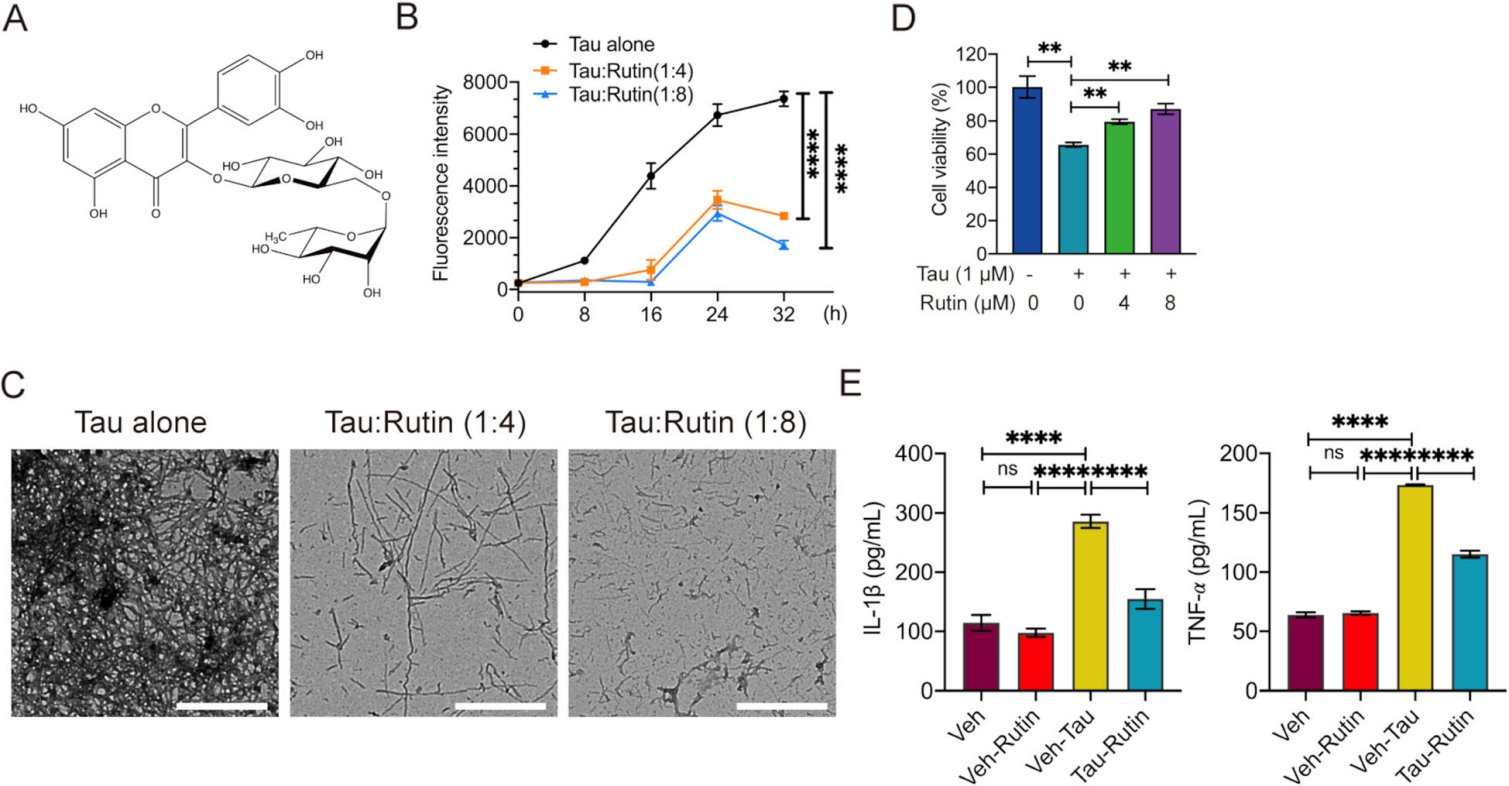
Rutin inhibits tau aggregation, reduces tau oligomer-induced cytotoxicity and proinflammatory cytokine production. **A** The chemical structure of rutin. **B** The aggregation kinetics of 10 μM tau was assessed by thioflavin T fluorescence assay with or without the incubation of 40 or 80 μM of rutin. **C** The morphologies of tau were examined using a Hitachi H7650 TEM (scale bars 10 μm). **D** The viability of SH-SY5Y cells was determined by MTT assay when challenged with 1 μM tau oligomers in the presence or absence of rutin. **E** The levels of IL-1β and TNF-α were detected in the supernatants of primary microglial cultures when challenged with 1 μM tau oligomers in the presence or absence of 8 μM rutin. Experiments **B**, **D**, **E** were performed three times with at least biological triplicates in each experiment. Data represent means ± SEM and were analyzed by two-way ANOVA with Bonferroni’s test (**B**) or one-way ANOVA with Tukey’s test (**D**, **E**). **P < 0.01, ****P < 0.0001. ns, not significant

To detect the effect of rutin on tau aggregation, we used ThT fluorescence assay to monitor the fibril formation of Tau in presence or absence of rutin (Fig. 1A). Tau proteins are aggregation-prone and show an expected nucleation-dependent polymerization process. After 48 h of incubation, tau alone assembled into a large amount of long fibrils, as detected by TEM (Fig. 1B, C), while coincubation with rutin reduced tau aggregation and fibril formation (Fig. 1B). The addition of 40 μM rutin led to a decreased population of fibrils, and 80 μM rutin induced the formation of numerous small and amorphous tau aggregates (Fig. 1C). We next determined the effect of rutin on tau-mediated cytotoxicity in SH-SY5Y cells. One μM tau oligomers reduced the cell viability to 65.7%, whereas rutin significantly increased the cell viability in a concentration-dependent manner (Fig. 1D).

### Rutin decreases microglial production of proinflammatory cytokines induced by tau oligomers

To investigate the mechanism by which rutin inhibits the neuroinflammation, we measured the levels of IL-1β and TNF-α in the supernatants of primary microglial cultures challenged with 1 μM tau oligomers in the presence or absence of 8 μM rutin for 24 h. The levels of IL-1β and TNF-α were significantly increased when the microglia cells were challenged by oligomeric tau, while rutin markedly decreased the production of IL-1β and TNF-α by 46% and 34%, respectively (Fig. 1E).

### Rutin protects neuronal morphology from toxic tau oligomers and promotes microglial engulfment of extracellular tau

**Fig. 2:**
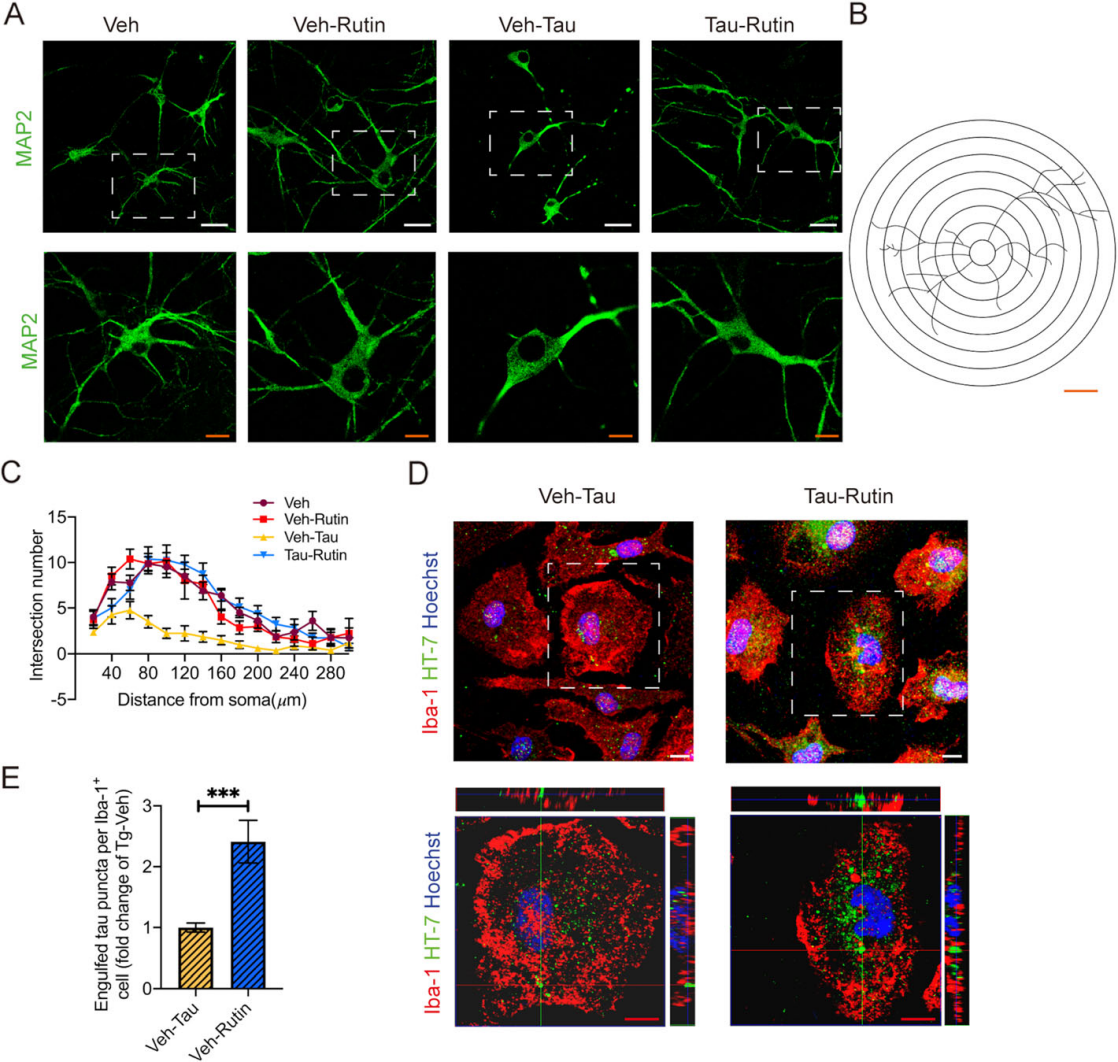
Rutin protects neuronal morphology from toxic tau oligomers and promotes microglial engulfment of extracellular tau. **A** Immunolabeling of MAP2 (green) in primary neuronal cultures treated with 1 μM tau oligomers in the presence or absence of 8 μM rutin for 24 h (scale bar: white, 25 μm; red, 10 μm). **B** The concentric rings spaced 20 μm apart centered on the soma center, which was used to count the number of intersections for the Sholl analysis. Scale bar: 40 μm. **C** Sholl analysis of the dendritic branching in primary neuronal cultures treated with 1 μM tau oligomers in the presence or absence of 8 μM rutin for 24 h. Forty neurons per culture from three independent cultures were used for the analysis. **D** Immunolabeling of tau (green) within Iba-1^+^ (red) cell in primary microglial cultures treated with 1 μM tau oligomers in the presence or absence of 8 μM rutin for 24 h (scale bar: 10 μm). **E** Quantification of tau puncta engulfed by Iba-1^+^ microglial cells in **D**. At least 20 microglial cells per culture from three independent cultures were used for the analysis. Data represent means ± SEM and were analyzed by two-way ANOVA with Bonferroni’s test (**C**) or Student’s *t* test (**E**). ***P < 0.001

Tau oligomers may alter neuronal morphology and induce neuronal functional changes [24]. To test whether rutin has protective effect on neuronal morphology, we added 8 μM rutin to primary neuronal cultures upon 1 μM tau oligomer challenge (Fig. 2A). Sholl analysis revealed that the neuronal dendritic arbors were destroyed when exposed to tau oligomers for 24 h, whereas rutin significantly protected neuronal morphology from tau oligomer-induced toxicity (Fig. 2B, C). To determine the effect of rutin on microglial uptake of extracellular tau oligomers, we added 8 μM rutin to the primary microglial cultures in presence of 1 μM tau oligomers, and analyzed the level of intracellular tau 24 h later. A significant higher level of internalized tau was observed in microglia cells treated with rutin compared with those of vehicle controls, suggesting rutin promoted microglial uptake of extracellular tau oligomers (Fig. 2D, E).

### Rutin rescues memory loss in Tau-P301S mice

**Fig. 3:**
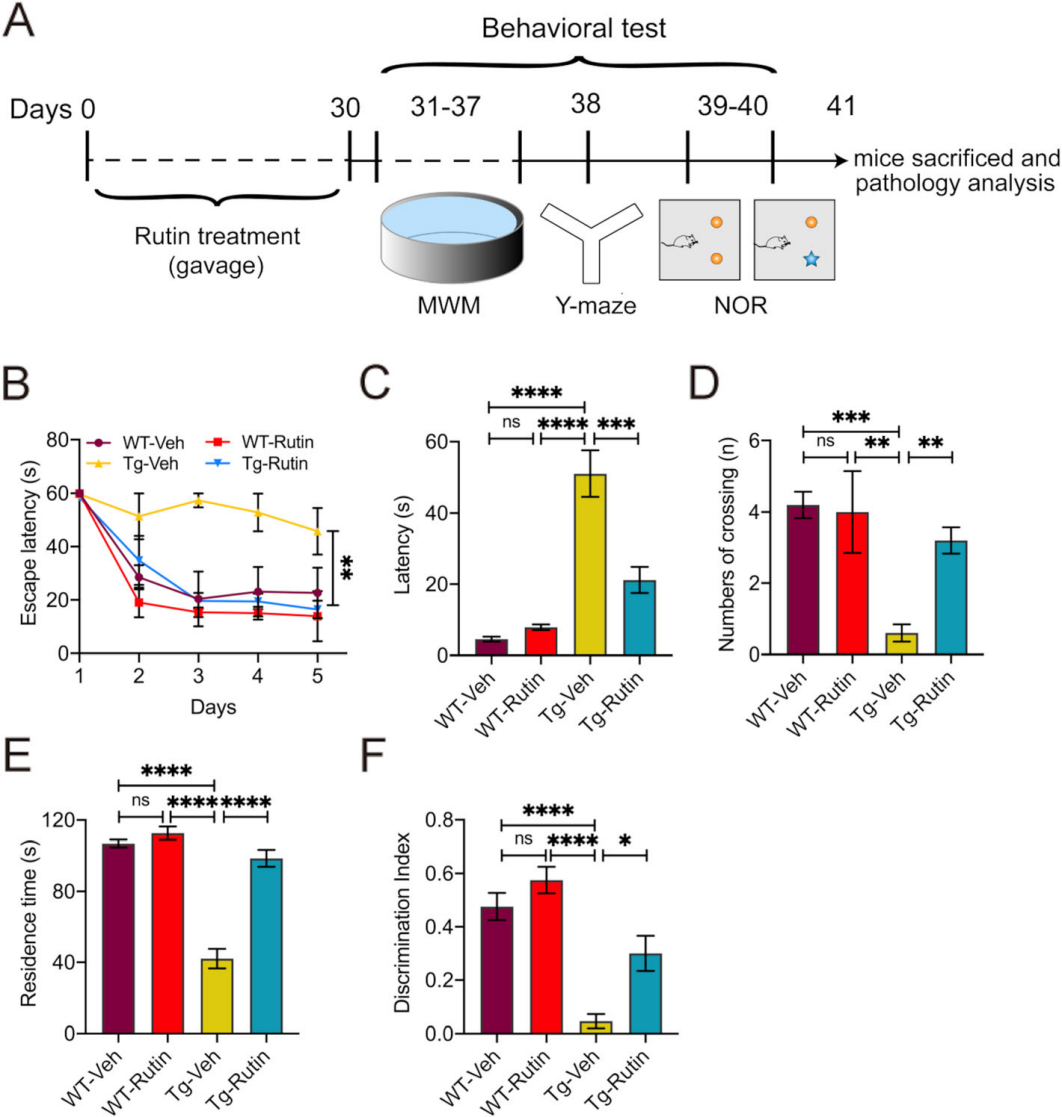
Rutin rescues memory deficits in Tau-P301S mice. **A** Schematic representation of pharmacological treatment and experimental measurement. **B**–**D** The Morris water maze was performed on Tau-P301S mice and their WT littermates treated with rutin or vehicle. (*n* = 8 mice). **B** The latency to find the hidden platform was measured during training trials. **C**, **D** During probe trials, the latency to the position of the removed platform (**C**) and the number of platform crossings (**D**) were measured. **E** The time spent in the novel arm in Y-maze test was determined on Tau-P301S mice and their WT littermates treated with rutin or vehicle (*n* = 8 mice). **F** The novel object recognition test was performed on Tau-P301S mice and their WT littermates treated with rutin or vehicle. The results were expressed as the discrimination index (*n* = 8 mice). Data represent means ± SEM and were analyzed by two-way ANOVA with Bonferroni’s test (**B**) or one-way ANOVA with Tukey’s test (**C**–**F**). *P < 0.05, **P < 0.01, ***P < 0.001, ****P < 0.0001, ns, not significant

To investigate the effect of rutin on memory and cognitive performance of Tau-P301S mice, we treated Tau-P301S mice with rutin daily for 30 days and performed the MWM, Y-maze, and object recognition tests thereafter (Fig. 3A). During the training period of MWM test, Tau-P301S mice treated with rutin showed improved memory retention by taking shorter time to reach the hidden platform compared with Tau-P301S mouse controls (Fig. 3B). Similarly, in probe trials, rutin-treated Tau-P301S mice exhibited markedly decreased escape latencies to the platform (Fig. 3C) and increased platform crossing number (Fig. 3D). In Y-maze test, Tau-P301S mice treated with rutin spent more time in the novel arm than vehicle-treated control mice (Fig. 3E). In NOR test, treatment with rutin significantly increased the preferences for the novel object in Tau-P301S mice (Fig. 3F). Moreover, no significant difference in the motor performance of each mouse was observed in any mouse group (Fig. S2), indicating that neither group was impaired in motility and exploratory activities. Together, these findings showed that rutin significantly rescued memory deficits in Tau-P301S mice.

### Rutin attenuates tau pathology in Tau-P301S mice

**Fig. 4:**
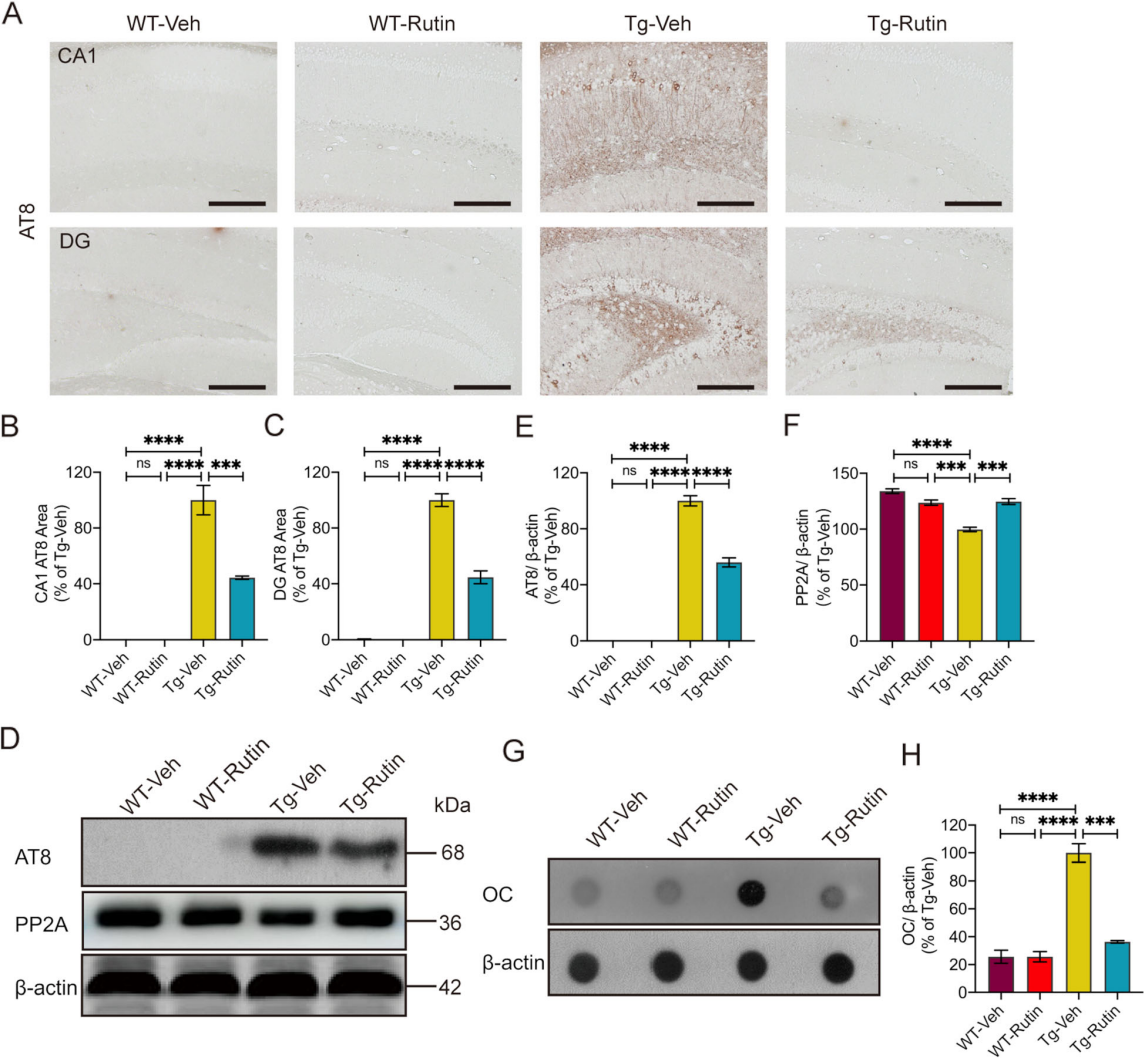
Rutin attenuates tau pathology in Tau-P301S mice. **A** AT8 immunostaining for phosphorylated-tau in the brains of Tau-P301S mice and their WT littermates treated with rutin or vehicle (scale bar: 100 μm). **B**, **C** Quantification of AT8 immunostaining in the hippocampal CA1 (**B**) and DG (**C**) regions (*n* = 8 mice). **D** Western blot analysis of phosphorylated-tau (AT8), PP2A, and β-actin in the brain lysates of Tau-P301S mice and their WT littermates treated with rutin or vehicle. **E**, **F** Quantification of AT8 positive p-tau (**E**) and PP2A (**F**) in **D** (*n* = 8 mice). **G** Dot-blot analysis of OC-positive oligomers in the brain lysates of Tau-P301S mice and their WT littermates treated with rutin or vehicle. **H** Quantification of OC-positive tau oligomers (*n* = 8 mice). Data represent means ± SEM and were analyzed by one-way ANOVA with Tukey’s test (**B**–**F**, **H**). ***P < 0.001, ****P < 0.0001, ns, not significant

To assess the effect of rutin on the tau pathology in Tau-P301S mice, we performed AT8 immunostaining for phosphorylated-tau (p-tau) in the brains of mice (Fig. 4A). A significant decreased level of tau hyperphosphorylation was detected in the hippocampal CA1 and dentate gyrus (DG) regions of rutin-treated Tau-P301S mice (Fig. 4B, C). Consistently, rutin significantly reduced p-tau levels in the brain lysates of Tau-P301S mice as detected by western blotting (Fig. 4D, E).

Given protein phosphatase 2A (PP2A) plays a critical role in regulating tau phosphorylation [25, 26], we determined the levels of PP2A in the brain lysates of Tau-P301S mice using western blotting. Decreased levels of PP2A were detected in Tau-P301S mice compared to WT mice. However, rutin significantly increased PP2A levels in Tau-P301S mice (Fig. 4D, F).

Tau oligomers are widely regarded as the most toxic and pathogenic form of tau [11, 27]. To assess the effect of rutin on the level of tau oligomers, we further determined the oligomer levels in mouse brain lysates by dot-blot using fibrillar oligomer-specific antibody OC. A 63.7% reduction of OC-positive tau oligomers was detected in rutin-treated Tau-P301S mice, as compared with the vehicle-treated controls (Fig. 4G, H, S1c).

### Rutin reduces neuroinflammation by downregulating NF-κB pathway in the brains of Tau-P301S mice

**Fig. 5:**
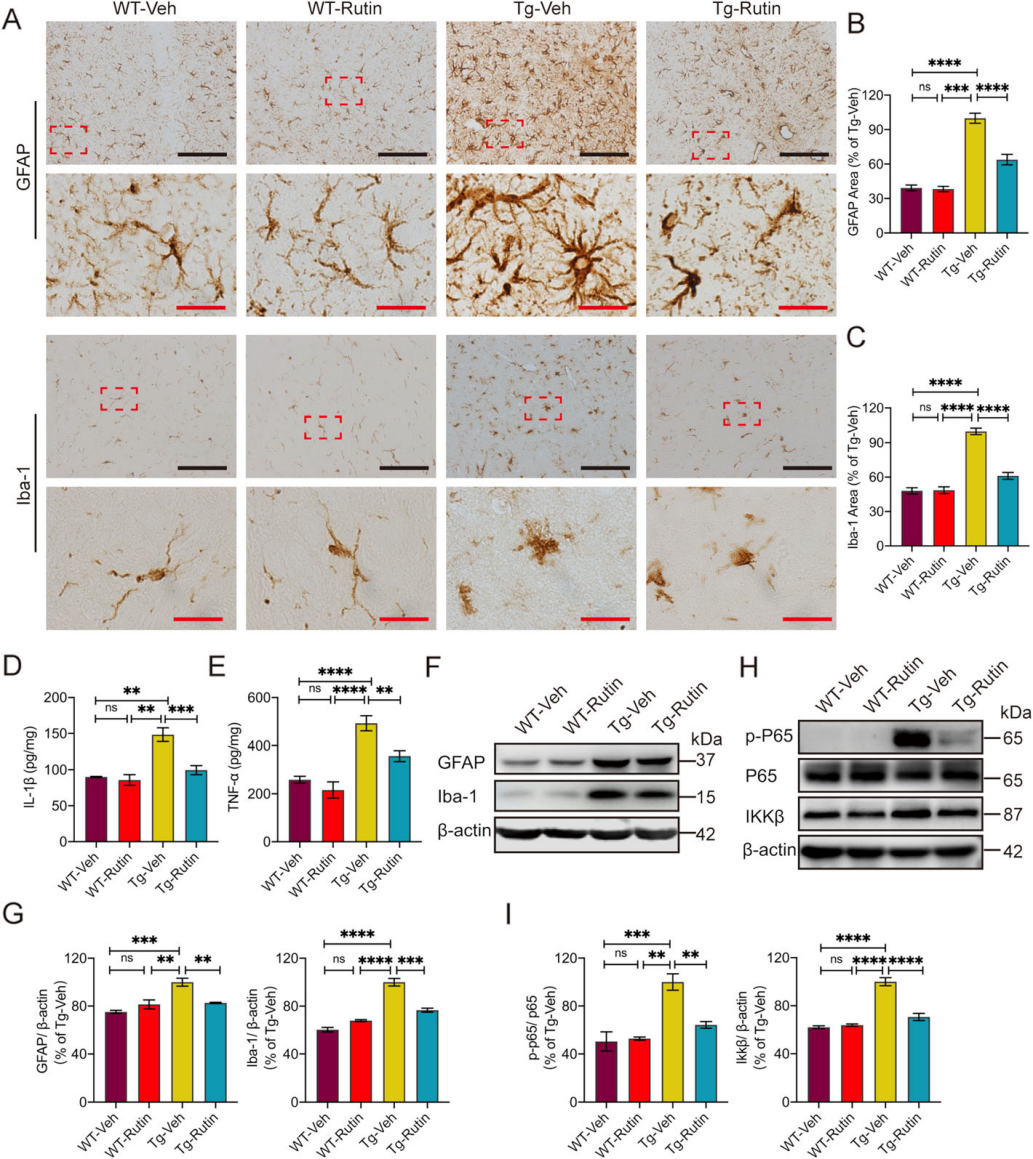
Rutin reduces neuroinflammation in the brains of Tau-P301S mice. **A** GFAP immunostaining and Iba-1immunostaining in the brains of Tau-P301S mice and their WT littermates treated with rutin or vehicle (scale bar: black, 100 μm; red, 10 μm). **B**, **C** Quantification of GFAP (**B**) and Iba-1 (**C**) immunostaining in **A**. (n = 8 mice). **D**, **E** The levels of IL-1β (**D**) and TNF-α (**E**) in the brain lysates of Tau-P301S mice and their WT littermates were determined using corresponding ELISA kits. (*n* = 8 mice). **F** Western blot analysis of GFAP, Iba-1, and β-actin in the brain lysates of Tau-P301S mice and their WT littermates treated with rutin or vehicle. **G** Quantification of GFAP and Iba-1 in **F**. (*n* = 8 mice). **H** Western blot analysis of p-P65, total P65, IKKβ, and β-actin in the brain lysates of Tau-P301S mice and their WT littermates treated with rutin or vehicle. **I** Quantification of p-P65/P65 and IKKβ in **H**. (*n* = 8 mice). Data represent means ±SEM and were analyzed by one-way ANOVA with Tukey’s test (**B**–**E**, **G**, **I**). **P < 0.01, ***P < 0.001, ****P < 0.0001, ns, not significant

Neuroinflammation plays a key role in the pathogenesis of tauopathy, which is characterized by extensive gliosis and the release of inflammatory cytokines [6, 28]. To assess inflammation-associated pathology in the brains of Tau-P301S mice, we evaluated astrogliosis and microgliosis by GFAP and Iba-1 immunostaining, respectively (Fig. 5A). Rutin significantly reduced the astrogliosis and microgliosis in the brains of Tau-P301S mice (Fig. 5B, C). Western blot analysis consistently showed the decreased levels of GFAP and Iba-1 in the brains of Tau-P301S mice treated with rutin (Fig. 5F, G). We also found the levels of IL-1β and TNF-α in the brains of Tau-P301S mice were higher than those of WT mice, whereas rutin markedly decreased the levels of these proinflammatory cytokines (Fig. 5D, E).

The nuclear factor-kappa B (NF-kB) activation is closely associated with AD via neuroinflammation [29, 30]. To investigate whether rutin influences the NF-kB pathway in the brains of Tau-P301S mice, we determined the levels of IKK-β, P65 and p-P65 in the brain lysates of mice by western blotting. A significant increase in IKK-β and p-P65/P65 ratio was observed in Tau-P301S mice relative to that of WT mice, while treatment with rutin markedly decreased the levels of IKK-β and p-P65/P65 in Tau-P301S mice (Fig. 5H, I).

### Rutin rescues synapse loss in Tau-P301S mice

**Fig. 6:**
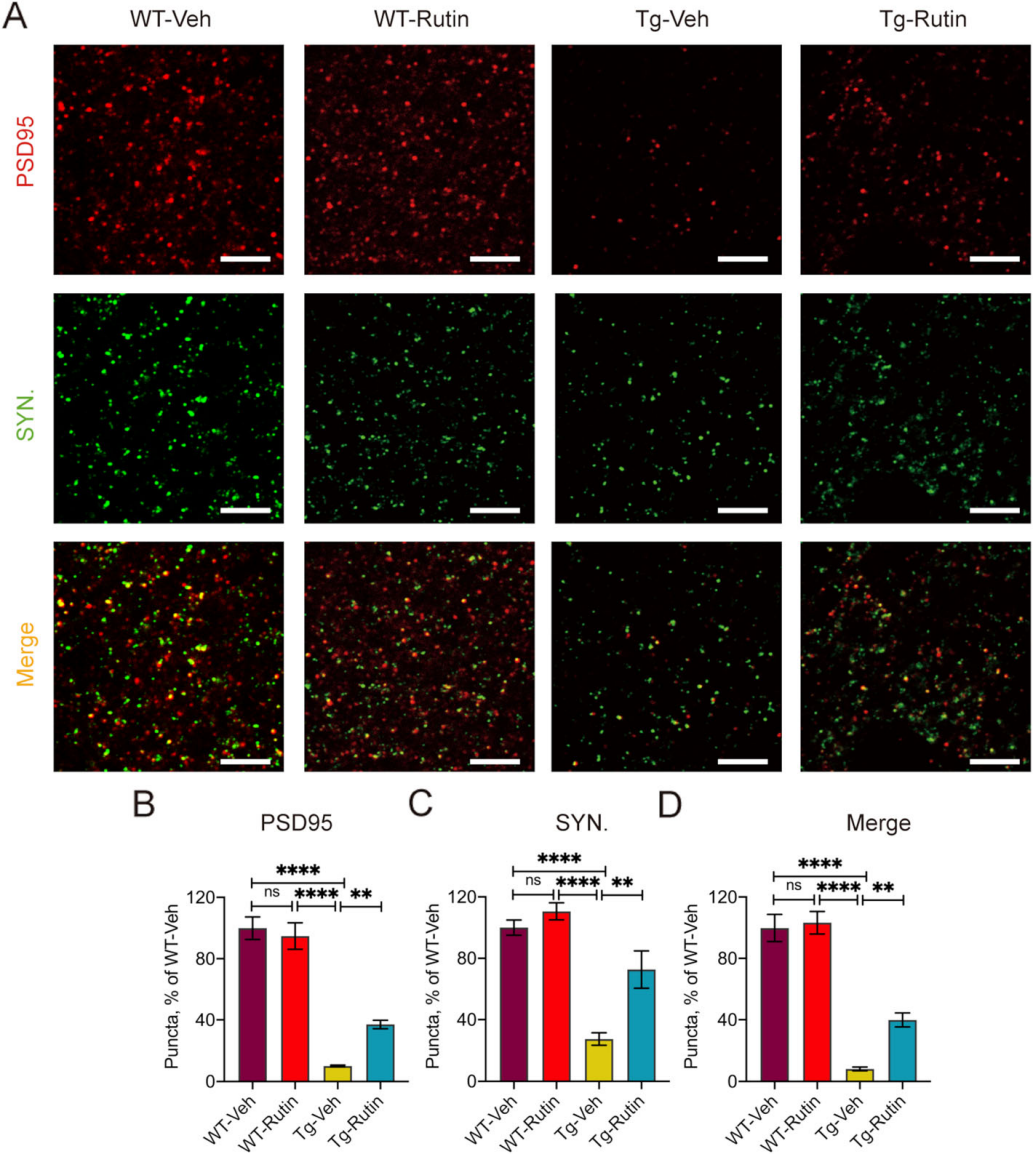
Rutin rescues synapse loss in Tau-P301S mice. **A** PSD95 immunostaining and synaptophysin immunostaining in the brains of Tau-P301S mice and their WT littermates treated with rutin or vehicle (scale bar: 3 μm). **B**–**D** Quantification of PSD95 puncta (**B**), synaptophysin puncta (**C**) and their apposition (**D**) in **A**. (*n* = 8 mice). Data represent means ± SEM and were analyzed by one-way ANOVA with Tukey’s test (**B**–**D**). **P < 0.01, ****P < 0.0001, ns, not significant

Synapse loss is a key feature of tauopathies, which is strongly linked to cognitive deficit [9, 10]. To assess the effect of rutin on synapse density in Tau-P301S mice, we measured the levels of two synaptic marker proteins, synaptophysin and postsynaptic density protein-95 (PSD95) in the brains of Tau-P301S mice by immunohistochemistry (Fig. 6A, S3). The colocalization of pre- and postsynaptic markers represent structural integrity of synapses, quantification of colocalized pre- and postsynaptic puncta (synaptophysin and PSD95) revealed a significant increase of synapses in Tau-P301S mice treated with rutin (Fig. 6B–D), suggesting that rutin rescued synapse loss in Tau-P301S mice.

### Rutin prevents microglial synapse engulfment in Tau-P301S mice

**Fig. 7:**
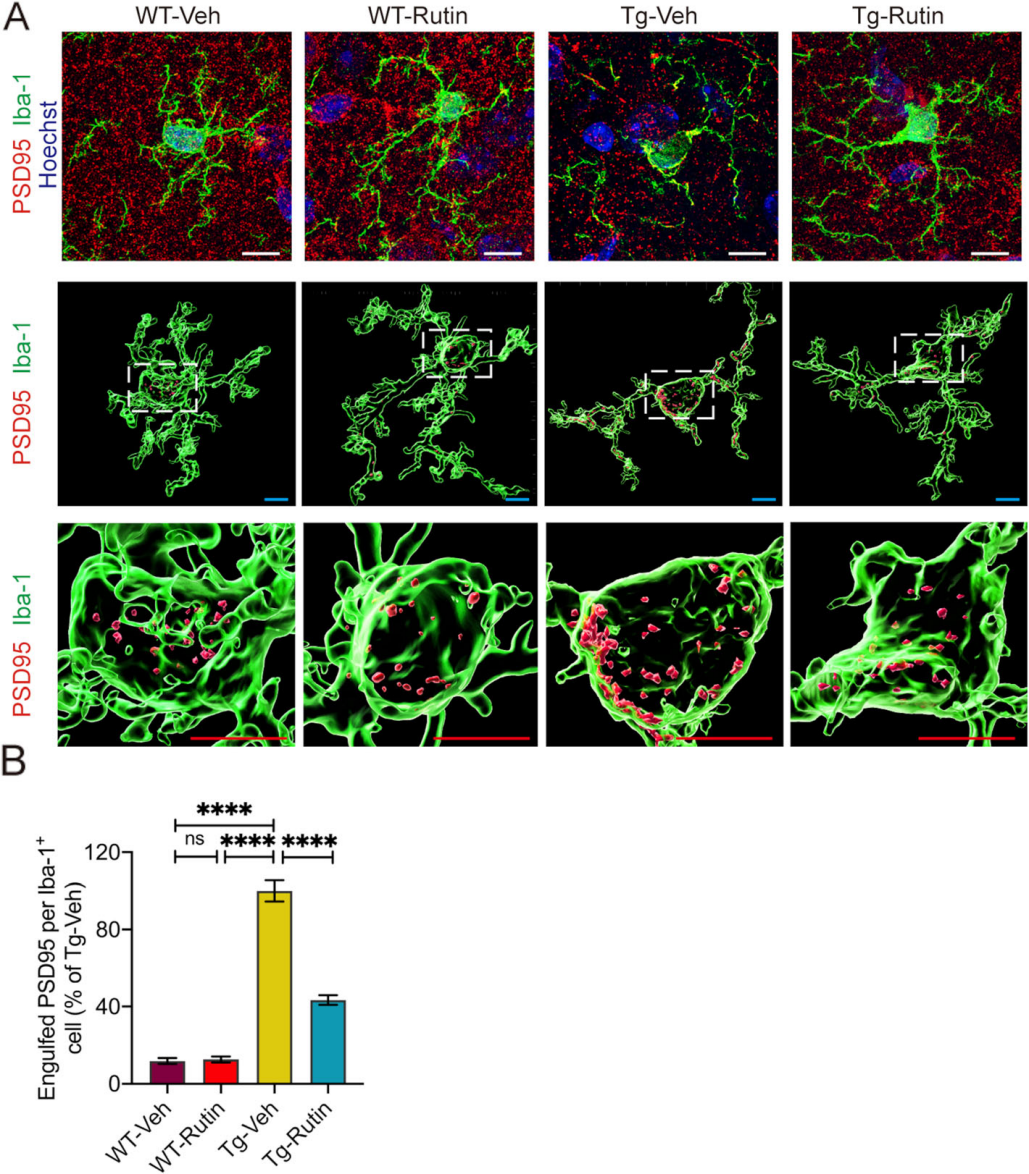
Rutin prevents microglial synapse engulfment in Tau-P301S mice. **A** Representative images show the engulfed PSD95 (red) puncta within Iba-1^+^ (green) microglial cells in the brains of Tau-P301S mice and their WT littermates treated with rutin or vehicle (scale bar: white, 10 μm; cyan, 5 μm; red, 5 μm). **B** Quantification of PSD95 puncta per Iba-1^+^ microglial cell (*n* = 8 mice). Data represent means ± SEM and were analyzed by one-way ANOVA with Tukey’s test. ****P < 0.0001, ns, not significant

To determine whether rutin can prevent microglial synapse engulfment to rescue synapse loss in vivo, we quantified PSD-95 puncta in microglia (Fig. 7A, S4). We observed PSD95 engulfment is highly present in microglia in Tau-P301S mice but not WT mice, whereas rutin treatment resulted in a 56.7% reduction of PSD95 puncta in microglia of Tau-P301S mice (Fig. 7B). Our results demonstrate that rutin reduces synapse engulfment by microglia and leads to a recovery of synapse density in Tau-P301S mice.

## Discussion

Alzheimer’s disease is a chronic, multifaceted and multifactorial neurodegenerative disorder. For more than three decades, the amyloid hypothesis proposes that Aβ is the driving force of AD, which triggers a deleterious cascade involving tau pathology and neurodegeneration [31]. Accumulating evidence now suggests that Aβ and tau pathologies have synergistic effects [32]. This may not only help explain the continued failure of anti-Aβ clinical trials but also suggest that rational strategy to counteract AD may need to target both pathologies simultaneously. Our previous study demonstrated that rutin reduced Aβ pathology and improved spatial memory in APP/PS1 mouse model [14]. We here reported that rutin rescued cognitive deficits and synapse loss, attenuated tau pathology and neuroinflammation in Tau-P301S mouse model. Our findings indicate that rutin is a promising drug candidate for AD treatment by combinatorial targeting of tau and Aβ.

Tau protein plays a critical role in the physiology and morphology of neurons [33]. In AD, excessive hyperphosphorylated tau appears to aggregate into intracellular NFTs, which correlates well with the onset of clinical symptoms [5]. Here, we observed that rutin inhibited tau aggregation, which is consistent with the effects of rutin on Aβ accumulation. The mechanism underlying rutin inhibits amyloid aggregation could be that its aromatic structure binds to the hydrophobic β-sheet secondary structure of amyloid aggregates and disturbs polymeric features of amyloidogenic proteins [21, 28, 34]. Misfolding of tau and its assembly into oligomers has been considered to contribute to tau-mediated toxicity [11]. As the most toxic species, tau oligomers lead to a deterioration of neuronal function, including synapse loss, oxidative stress, and impairment of axonal transport and mitochondrial function. Our present study revealed that rutin markedly prevented tau oligomer-induced cytotoxicity and protected neuronal morphology. In pathological conditions, tau aggregates could be released from neurons into extracellular space and be taken up by other neurons, leading to tau propagation and the spread of tau pathology [35, 36]. Here in cultured primary microglia, we found that rutin treatment significantly promoted microglial engulfment of extracellular tau oligomers, thus blocking the spread of tau pathology within neurons.

Genetic and molecular insights into the pathophysiology of AD reveal that hyperphosphorylation of tau plays an important role in AD progress [37]. Hyperphosphorylation is crucial for tau to detach from microtubules, which increases cytoplasmic tau levels, and causes tau to aggregate into oligomers [8, 38]. In our study, rutin significantly reduced both levels of p-tau and OC-positive tau oligomers. PP2A accounts for approximately 70% of the total tau phosphatase activity in human brains [26], and expression of PP2A is significantly decreased in the brains of AD patients [25]. We here showed that rutin treatment elevated PP2A levels in the brains of Tau-P301S mice, which contributing to a significant improvement of cognitive performance in Tau-P301S mice.

Rutin has been reported to exert various biological effects, which mainly links to its anti-inflammatory activity [14, 15, 21]. Neuroinflammatory processes are strongly associated with tau pathology and play an important role in the development and progression of AD [6]. Our results showed that rutin directly lowered microglial production of proinflammatory cytokines mediated by tau oligomers in vitro. Rutin treatment significantly reduced neuroinflammation through decreasing gliosis and the levels of IL-1β and TNF-α in the brain lysates of Tau-P301S mice. The anti-inflammatory mechanism of rutin was the downregulation of the abnormal NF-κB activation.

Synapse loss and tau pathology correlate with cognitive impairment, which are hallmarks of AD [39]. During AD development, pathological tau accumulates and mislocalizes to synapses, leading to synaptic dysfunction and synapse loss [9, 10]. Moreover, increased microglial engulfment of synapses was found in AD patients and transgenic mice, correlating with a drop in synapse density [9]. Our results revealed that rutin significantly prevented microglial synapse engulfment of synapses and restored the levels of PSD95 and synaptophysin, thus rescuing synapse loss in the brains of Tau-P301S mice.

Rutin presents as a natural flavonoid with a wide range of biological activities. In the present study, our results indicate that rutin exert multiple effects simultaneously. Rutin inhibits tau aggregation and tau oligomer-induced cytotoxicity, lowers the production of proinflammatory cytokines, protects neuronal morphology from toxic tau oligomers, and promotes microglial uptake of extracellular tau oligomers in vitro. Based on these mechanisms, rutin reduced tau pathology, regulated tau hyperphosphorylation by increasing PP2A level in vivo, inhibited neuroinflammation via normalizing of NF-κB pathway, prevented microglial synapse engulfment and rescued synapse loss in the brains of Tau-P301S mice.

Rutin has been reported to exert various neuroprotective effects. Although the poor water solubility and low bioavailability, rutin is able to pass the blood brain barrier (BBB). It is reported that the peak plasma concentration (Cmax) of rutin was 262.85 ± 6.15 ng/ml after oral administration at a dose of 35 mg rutin to rabbits [40], and the concentration of rutin in serum was about 300 ng/ml after 2 h of oral administration with 100 mg/kg rutin to mice [19]. After i.v. administration to rats at the dose of 10 mg/kg, the Cmax of rutin in plasma and brain homogenate was 1511.24 ± 46.92 ng/ml and 111.57 ± 12.01 ng/ml, respectively [41]. Recently, various strategies have been developed to improve its solubility and bioavailability in order to extend its clinical application. A sodium salt of rutin has been reported to be highly water soluble and bioavailable, with increased ability for BBB penetration [19]. In our study, we found that rutin exerted multiple effects on pathological tau in vitro. In AD patients, the concentrations of total-tau in CSF were about 700 pg/ml [42]. Although the low bioavailability, rutin can pass BBB and reach the CSF concentrations high enough to neutralize pathological tau. Therefore, we believe rutin should exert direct effects on tau pathology in the brain rather than indirect affecting the brain pathology by effects on gut microbiota or cardiovascular function.

## Conclusions

Given Aβ-tau synergy in AD pathogenesis, the combinatorial targeting of tau and Aβ would largely improve treatment efficacy. Based on rutin’s therapeutic effects on Aβ and tau pathology and its good safety profile, rutin presents as a promising drug candidate for AD treatment.

## Supplementary Information

**Additional file 1:.** Fig.S1 Tau oligomer preparation and control dot-blots of brain lysates without primary antibody OC. a Tau oligomer preparation by size exclusion chromatography. b Oligomer native gel electrophoresis visualized by coomassie stain, followed by western blot using HT-7 antibody. c Control dot-blots of brain lysates were probed without primary antibody OC, or using β-actin as a control. The blots were stained for total protein with Ponceau S simultaneously. Fig.S2. The motor performance of mice in behavioral test. a The travelled distance in probe trial of MWM. b The swim speed in MWM. c The travelled distance in Y-maze. d The velocity of mice in Y-maze. e The travelled distance in NOR. f The total exploration time in NOR. Fig.S3 Rutin rescues synapse loss in Tau-P301S mice. PSD95 immunostaining and synaptophysin immunostaining in the brains of Tau-P301S mice and their WT littermates treated with rutin or vehicle. (Scale bar: 10 μm). Fig.S4 Rutin prevents microglial synapse engulfment in Tau-P301S mice. Representative images show the engulfed PSD95 (red) puncta within Iba-1^+^ (green) microglial cells in the brains of Tau-P301S mice and their WT littermates treated with rutin or vehicle. (Scale bar: cyan, 25 μm; white, 20 μm).

## Data Availability

The datasets used and/or analyzed during the current study are available from the corresponding author on reasonable request.

## Abbreviations

AD: Alzheimer’s disease
ANOVA: Analysis of variance
CMC: Carboxymethylcellulose
DAB: Diaminobenzidine
DG: Dentate gyrus
DMEM: Dulbecco’s modified Eagle’s medium
DMSO: Dimethyl sulfoxide
FBS: Fetal bovine serum
GFAP: Glial fibrillary acidic protein
Iba-1: Ionized calcium-binding adapter molecule 1
IGF1: Insulin-like growth factor 1
IL-1β: Interleukin 1β
MAP 2: Microtubule associated protein 2
MTT: 3-(4,5-Dimethylthiazol-2-yl)-2,5diphenyltetrazolium bromide
MWM: Morris water maze
NF-κB: Nuclear factor κB
NFTs: Neurofibrillary tangles
NOR: Novel objection recognition
PP2A: Phosphatase 2A
PSD95: Postsynaptic density protein-95
TEM: Transmission electron microscopy
ThT: Thioflavin T
TNF-α: Tumor necrosis factor α
