# Supplementary material for: Rutin prevents tau pathology and neuroinflammation in a mouse model of Alzheimer’s disease
Source: J Neuroinflammation. 2021 Jun 11;18:131. doi: 10.1186/s12974-021-02182-3 (PMC8196535; doi:10.1186/s12974-021-02182-3)
Supplement: Supplementary file 1 — Additional file 1:. Fig.S1 Tau oligomer preparation and control dot-blots of brain lysates without primary antibody OC. a Tau oligomer preparation by size exclusion chromatography. b Oligomer native gel electrophoresis visualized by coomassie stain, followed by western blot using HT-7 antibody. c Control dot-blots of brain lysates were probed without primary antibody OC, or using β-actin as a control. The blots were stained for total protein with Ponceau S simultaneously. Fig.S2. The motor performance of mice in behavioral test. a The travelled distance in probe trial of MWM. b The swim speed in MWM. c The travelled distance in Y-maze. d The velocity of mice in Y-maze. e The travelled distance in NOR. f The total exploration time in NOR. Fig.S3 Rutin rescues synapse loss in Tau-P301S mice. PSD95 immunostaining and synaptophysin immunostaining in the brains of Tau-P301S mice and their WT littermates treated with rutin or vehicle. (Scale bar: 10 μm). Fig.S4 Rutin prevents microglial synapse engulfment in Tau-P301S mice. Representative images show the engulfed PSD95 (red) puncta within Iba-1+ (green) microglial cells in the brains of Tau-P301S mice and their WT littermates treated with rutin or vehicle. (Scale bar: cyan, 25 μm; white, 20 μm). [file 12974_2021_2182_MOESM1_ESM.docx]

**Supplementary Materials for**

**Rutin prevents tau pathology and neuroinflammation in a mouse model of Alzheimer’s disease**

Xiao-ying Sun^1,3^†, Ling-jie Li^1,3^†, Quan-Xiu Dong^1,3^, Jie Zhu^1,2^, Ya-ru Huang^1,3^, Sheng-jie Hou^1,3^, Xiao-lin Yu^1,2*^, Rui-tian Liu^1,2*^

*Corresponding author

Rui-tian Liu

Email: rtliu@ipe.ac.cn;

Or Xiao-lin Yu

Email: yuxiaolin@ipe.ac.cn;


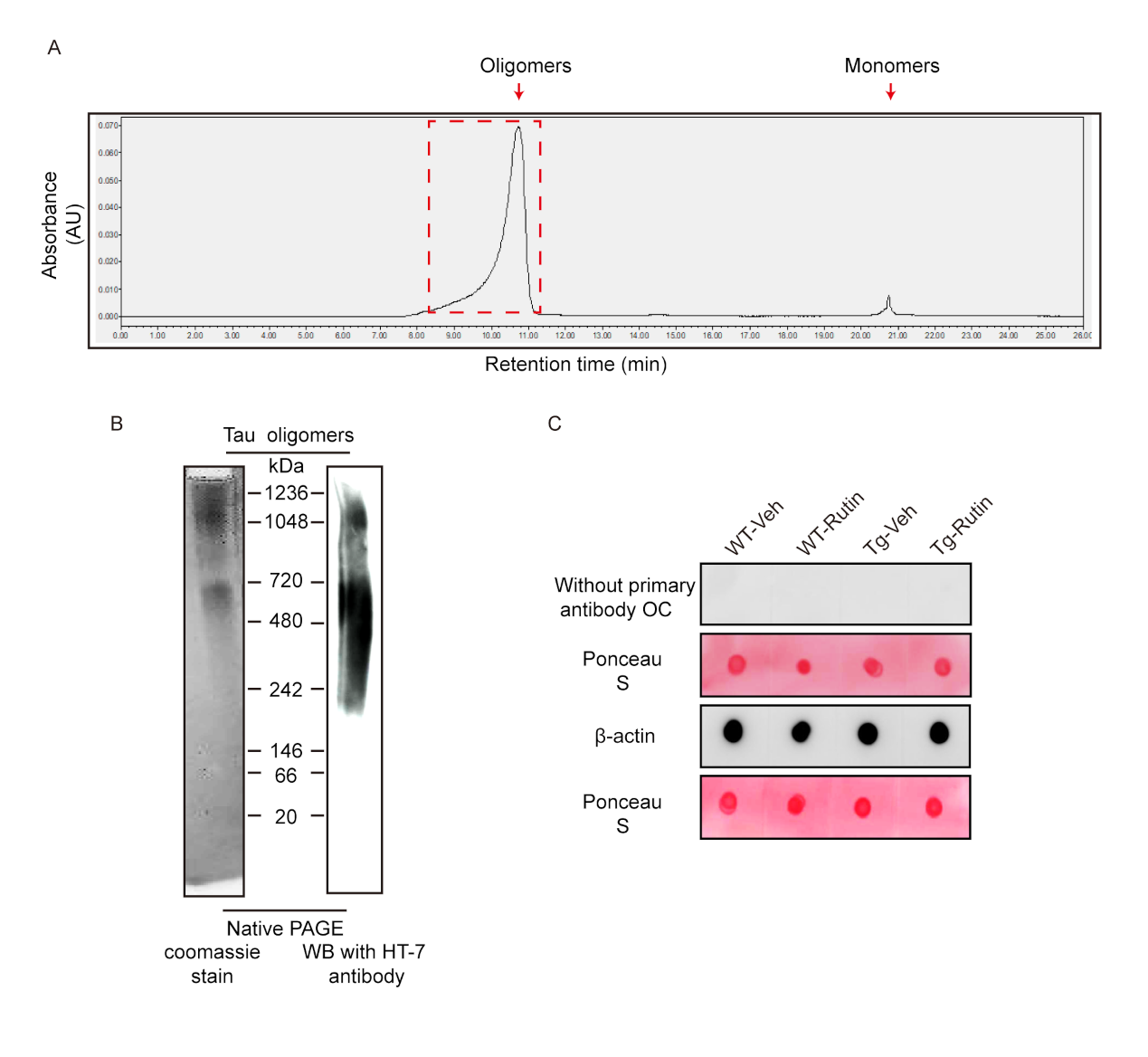


**Fig.S1 Tau oligomer preparation and control dot-blots of brain lysates without primary antibody OC.** **a** Tau oligomer preparation by size exclusion chromatography. **b** Oligomer native gel electrophoresis visualized by coomassie stain, followed by western blot using HT-7 antibody. **c** Control dot-blots of brain lysates were probed without primary antibody OC, or using β-actin as a control. The blots were stained for total protein with Ponceau S simultaneously.


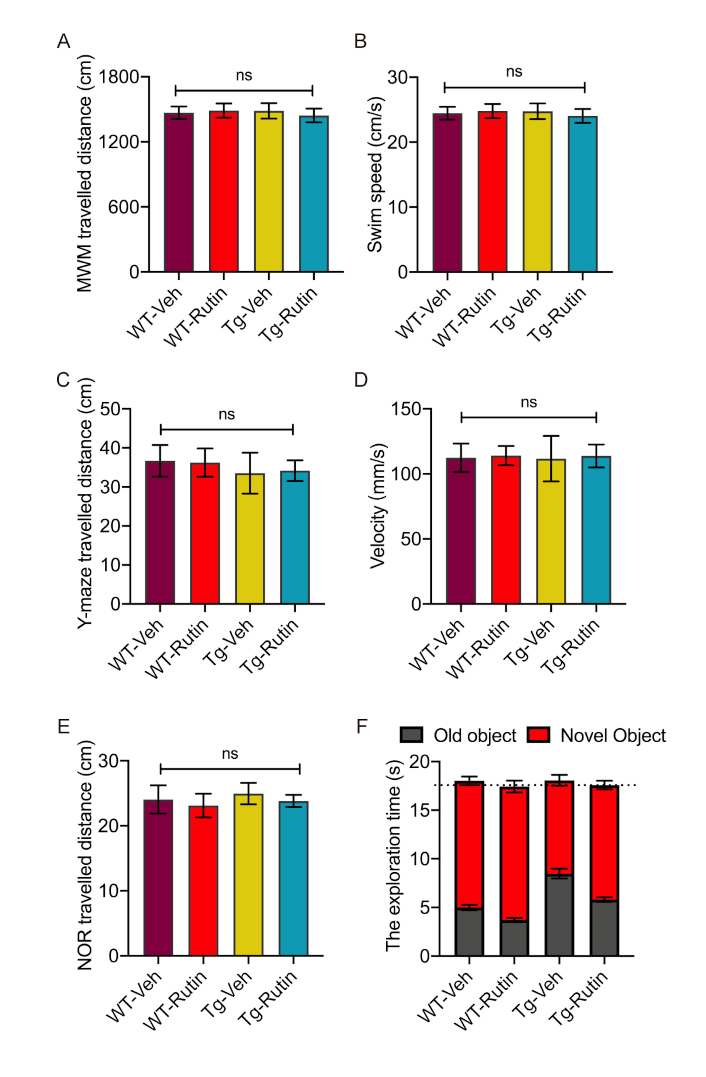


**Fig.S2. The motor performance of mice in behavioral test. a** The travelled distance in probe trial of MWM. **b** The swim speed in MWM. **c** The travelled distance in Y-maze. **d** The velocity of mice in Y-maze. **e** The travelled distance in NOR. **f** The total exploration time in NOR.


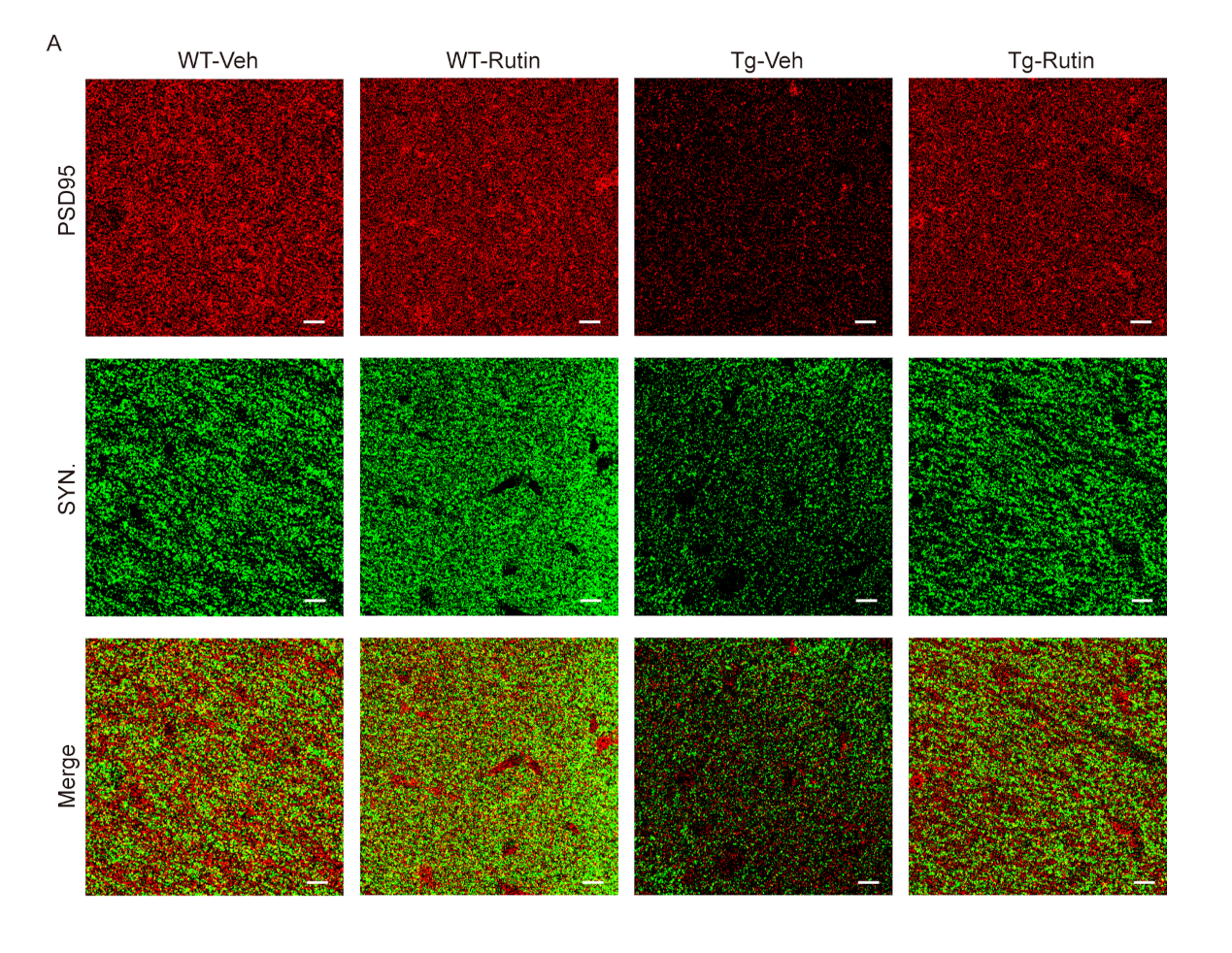


**Fig.S3 Rutin rescues synapse loss in Tau-P301S mice.** PSD95 immunostaining and synaptophysin immunostaining in the brains of Tau-P301S mice and their WT littermates treated with rutin or vehicle. (Scale bar: 10 μm).


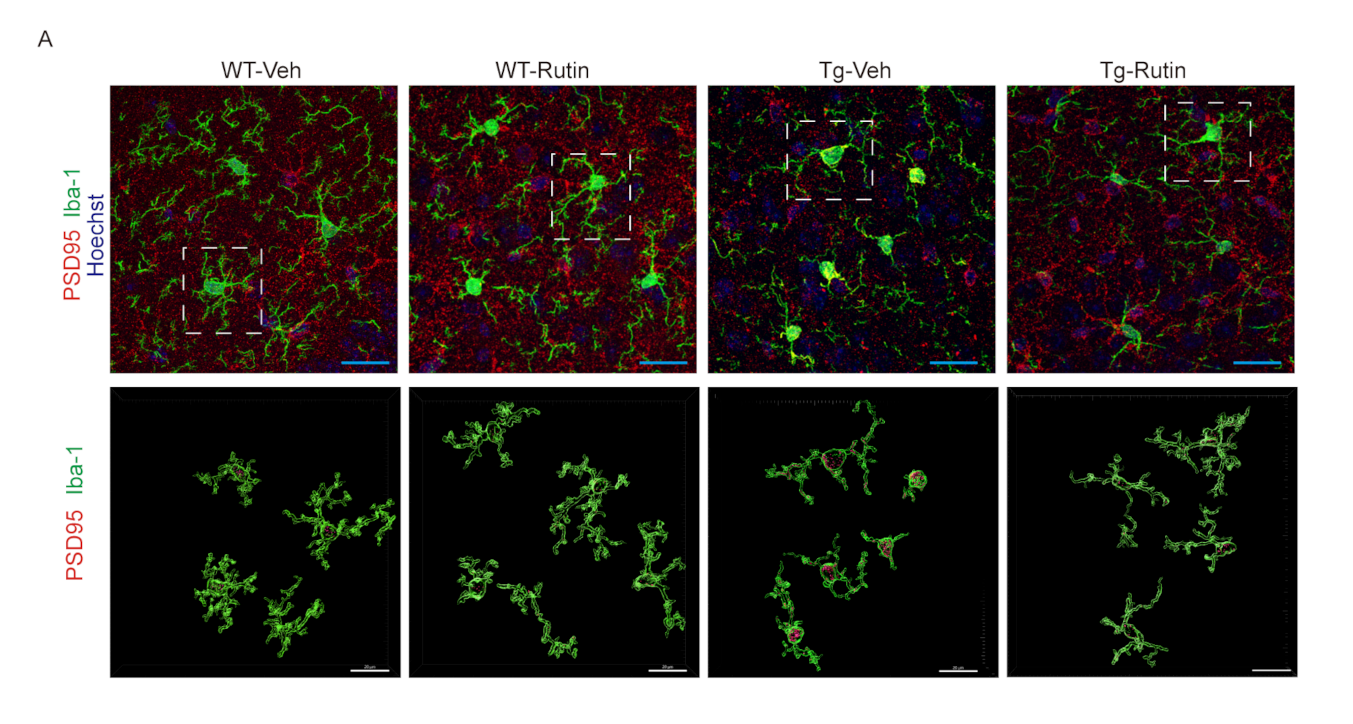


**Fig.S4 Rutin prevents microglial synapse engulfment in Tau-P301S mice.** Representative images show the engulfed PSD95 (red) puncta within Iba-1^+^ (green) microglial cells in the brains of Tau-P301S mice and their WT littermates treated with rutin or vehicle. (Scale bar: cyan, 25 μm; white, 20 μm).
